# Enhanced Detection of Visual-Evoked Potentials in Brain-Computer Interface Using Genetic Algorithm and Cyclostationary Analysis

**DOI:** 10.1155/2007/28692

**Published:** 2007-09-11

**Authors:** Cota Navin Gupta, Ramaswamy Palaniappan

**Affiliations:** Department of Computing and Electronic Systems, University of Essex, Wivenhoe Park, Colchester CO4 3SQ, UK

## Abstract

We propose a novel framework to reduce background electroencephalogram (EEG) artifacts from multitrial visual-evoked potentials (VEPs) signals for use in brain-computer interface (BCI) design. An algorithm based on cyclostationary (CS) analysis is introduced to locate the suitable frequency ranges that contain the stimulus-related VEP components. CS technique does not require VEP recordings to be phase locked and exploits the intertrial similarities of the VEP components in the frequency domain. The obtained cyclic frequency spectrum enables detection of VEP frequency band. Next, bandpass or lowpass filtering is performed to reduce the EEG artifacts using these identified frequency ranges. This is followed by overlapping band EEG artifact reduction using genetic algorithm and independent component analysis (G-ICA) which uses mutual information (MI) criterion to separate EEG artifacts from VEP. The CS and GA methods need to be applied only to the training data; for the test data, the knowledge of the cyclic frequency bands and unmixing matrix would be sufficient for enhanced VEP detection. Hence, the framework could be used for online VEP detection. This framework was tested with various datasets and it showed satisfactory results with very few trials. Since the framework is general, it could be applied to the enhancement of evoked potential signals for any application.

## 1. INTRODUCTION AND MOTIVATION

Oscillating potentials derived from the scalp surface using electrodes and believed to originate from outer layer of brain (neurons in the cortex) are called visual-evoked potential (VEP) signals [1]. These signals are derived from the brain's response to visual stimulation and have applications in numerous neuropsychological studies [1]. However, a major hurdle in analysing VEP, which is considered as a subset of event-related potential (ERP), is the extremely poor signal-to-noise ratio (SNR) of the VEP signals embedded within the ongoing background electroencephalogram (EEG). Averaging is commonly used to reduce the effects of EEG because VEP signals are assumed to be loosely time-locked to the stimulus, thereby adding up with averaging while EEG will be reduced due to its random property [2]. It is known that ERP is not a homogeneous signal, but instead a combination of different components due to which variations in amplitude and latency between trials are caused. Also, identical stimuli do not necessarily evoke identical responses [3, 4]; trial-to-trial variability can be appreciable, and ERP waveform, amplitude, and latency can change appreciably with time [3, 4]. Therefore, average ERP does not elicit the valid estimate of the VEP components amplitude and shape and hence is usually considered biased [4]. Next, the assumption that background EEG noise is random and uncorrelated seems untrue. Research has shown that EEG is not entirely uncorrelated with event-related activity [5]. Hence, the basic assumptions underlying signal averaging is generally violated with the above discussion.

ERPs consist of exogenous and endogenous components [6]. Exogenous components are obligatory responses which result on the presentation of physical stimuli. The endogenous components (say P300 component of the ERP signal) manifest the processing activities which depend on the stimuli's role within the task being performed by the subject [7]. P300-based brain-computer interface (BCI) systems [8–10] usually control the variance of the endogenous components. However, latency jitters are likely to affect endogenous VEP components more than exogenous components because variations due to cognitive process will affect the latencies of endogenous components that are less time locked to the event onset and are more dependent on the task [5]. It can, therefore, be problematic to compare the amplitudes of ERPs computed over trials with varying latency jitter [10]. These facts seem to question the validity of using the average ERP for clinical analysis; but however these issues are the main motivations for this work.

Techniques to improve conventional averaging like the Woody's method [11] have been proposed. In Woody's method individuals, trials are time shifted to compensate for latency shifts which are assumed to occur uniformly over the entire trial. However, this time-consuming technique's validity decreases when numerous iterations are used and it might not be the optimal solution under conditions of very low SNR [12]. A simple lowpass filter with a predetermined passband may improve SNR but may not necessarily provide an optimal separation of signal from noise in overlapping spectral ranges for all subjects under different experimental conditions. Weiner filter may be considered but the approach was devised for uncorrelated stationary signals with known spectra [4]. Also, the procedure for estimating filter weights, when the entire ERP epoch is used, has to strike a balance between short duration latency (i.e., sensory evoked) and large long duration (i.e., P300) components [4]. The differing power spectra do not make the resulting filter optimal for either type of components. Since these requirements are not met in ERP analysis, the optimality of Wiener filter is questionable.

Independent component analysis (ICA) has been extensively used for removal of artifacts from EEG data [13] as well as for analysis and detection of VEP signals [9, 14, 15]. However, [14, 15] also highlight the inherent limitations of ICA: first, VEP is assumed to be completely independent of the ongoing EEG. Temporal independence is not satisfied when training dataset is too small. In [9], ICA was used to separate P300 source from background EEG and it required a matched filter to be constructed uniquely for each subject. It uses a scheme similar to averaging for the identification of letters. ERP classification procedures proposed recently [16–18] are unsuitable for online implementation because none of them actually identify embedded variable ERP waveforms. In this paper, we present an alternative framework to enhance VEP detection by first identifying the embedded variable VEP frequency bands (which are highly masked by the background EEG activity) using cyclostationary analysis (CS). This allows us to remove the nonoverlapping frequency bands between VEP and EEG which increases the independence between background EEG artifacts and VEP signals for the genetic algorithm and independent component analysis module (G-ICA). Cyclostationary algorithm which is used in this paper has applications in many areas, for example, blind channel identification and equalisation [19], rotating machine monitoring [20], filter bank optimisation [21], and system identification [22]. This property has been used in the past for many communication applications [23, 24] and is the result of the implicit periodicity of these signals related to the baud rate, carrier frequency, or any other periodic component.

We then use a variation of our previous genetic algorithm (GA) work [25] to remove in-band EEG artifacts. Basic principles of ICA were used in the work. The G-ICA idea with kurtosis maximisation proposed in [26] was applied to denoise heart (ECG) signals in our recent study [25]. GA is a computational model inspired by evolution which may be used to solve search and optimisation problems and is a form of artificial intelligence. The basic approach creates a population of chromosomes, which are a string of values representing potential solutions to a problem. Through the theory of natural selection and genetic recombination, these solutions evolve into future populations where only the important combinations of chromosomes survive. The ability to investigate many possible solutions simultaneously is the main advantage of GA [27]. GA minimises the mutual information (MI) criterion [28], the fitness function used in this work to separate EEG artifacts from VEP signals. MI measures general statistical dependence between variables and is invariant to monotonic transformations performed on the variables. The G-ICA method is simpler when compared to the ICA because it does not require complex neural learning algorithms [25]. We apply the proposed framework to enhance the detection of P300 components for BCI design.

## 2. METHODOLOGY

**Figure 1:**
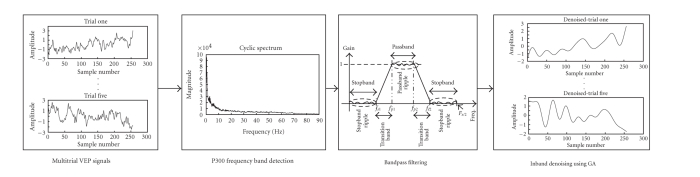
Block diagram of the proposed approach for use in VEP-based BCI design.

The novel framework to reduce background EEG artifacts from multitrial VEP signals for use in BCI design is shown diagrammatically in Figure 1. This scheme exploits the intertrial similarities of the VEP components in the frequency domain using CS analysis and removes the in-band EEG artifacts using G-ICA. This scheme overcomes the latency distortions of the many techniques proposed so far to detect the endogenous VEP components.

### 2.1. Cyclostationary analysis for VEP band detection

#### 2.1.1. Theory

We briefly discuss the theory of cyclostationary signals. A discrete-time signal which has periodic mean and correlation is said to be cyclostationary [29]. In particular, a signal *x*(*t* is called first-order cyclostationary [30] if its time-varying mean *m*_*x*_ = *E*[*x(t)*] is periodic:

(1) $$m_{x}(t+lp_{1})=m_{x}(t)\;\;\forall t,l\in Z.$$

Similarly, *x* is second-order cyclo-stationary [30] if its time-varying correlation

(2) $$R_{xx}(t;\tau )=E[x(t)x(t+\tau )]$$

is periodic in *t* for any fixed *τ* :

(3) $$R_{xx}(t+lp_{2};\tau )=R_{xx}(t;\tau )\;\;\forall t,l\in Z.$$

Here, *p*_1_ and *p*_2_ are the smallest positive integers such that (1) and (3) hold, respectively. If *p*_1_ and *p*_2_ = 1, we observe from (1) and (3) that mean is time invariant and the correlation depends on the time difference only. Then, *x*(*t*) is considered as a stationary signal or in the given discussion context a cyclostationary signal with period of one.

In the frequency domain cyclostationary (CS) analysis, if *x*(*t*) considered in the above discussion is cyclostationary and has a time period *T*_°_ or fundamental frequency *f*_°_(= 1/*T*_°_). We can define cyclic autocorrelation function of the same signal as follows [23]:

(4) $$R_{xx}(\tau ,f)=E\{x(t)x(t+\tau )\exp(-j2\pi ft)\}.$$

On averaging the various lags of the cyclic autocorrelation in frequency domain (4), we obtain a cyclic spectrum. The cyclic autocorrelation function (4) also satisfies the following property:

(5) $$R_{xx}(\tau ,f)=\{\begin{array}{ll} \text{finite} & \text{if}f=nf_{o},\\ 0, & \text{otherwise}, \end{array}$$

where *n* is a nonzero integer. In the frequency domain, cyclostationary processes are characterized by the cyclic spectrum, which represents the density of correlation between the spectral components of a process which are separated by an amount equal to the cycle frequency. The frequency components in a stationary signal are not correlated with each other so the cyclic autocorrelation of a stationary signal which is not cyclostationary is zero for all values of *f*, except *f* = 0 [31].

#### 2.1.2. Cyclo model for signal analysis

Based on the cyclostationary literature [23, 31], we discuss the cyclo model for signal analysis. Consider any recorded signal *x*(*t*) obtained by corrupting the clean signal *s*(*t*) with an additive noise signal *n*(*t*) as below [31]:

(6) x(t)=s(t)+n(t).

The noise *n*(*t*) is assumed to be nonperiodic with any statistical distribution.

Let *R*_*x*_(*t,f*), *R*_*s*_(*t,f*) *R*_*n*_(*t,f*) be the cyclic autocorrelation functions of *x* *(t)*, *s* *(t)* and *n* *(t)*, respectively. We can then write (6) in cyclic autocorrelation domain as [31]:

(7) $$R_{x}(\tau ,f)=R_{s}(\tau ,f)+R_{n}(\tau ,f).$$

Since *n* *(t)* is not cyclostationary, it means that *R*_*n*_(*t,f*) = 0, for *f* ≠ 0 and (7) becomes

(8) $$R_{x}(\tau ,f)=R_{s}(\tau ,f)\;\text{for}f\neq 0.$$

This model suggests that, independent of noise statistics, the cyclic autocorrelation function is insensitive to noise as long as the noise is not periodic.

#### 2.1.3. VEP signal band detection using cyclostationary analysis

We present a scheme based on the above model for enhanced detection of VEP band by exploiting the cyclostationarity property. The salient feature of this technique is the fact that trials are not required to be phase locked when recorded. To exploit the intertrial similarities of the VEP signal components in the frequency domain, cyclostationary is introduced by concatenating the recorded trials. The periodic repetition of the P300 components in the VEP trials for all trials (i.e., 300–600 ms after the occurrence of stimuli) enables cyclic analysis of the VEP signals.

**Figure 2:**
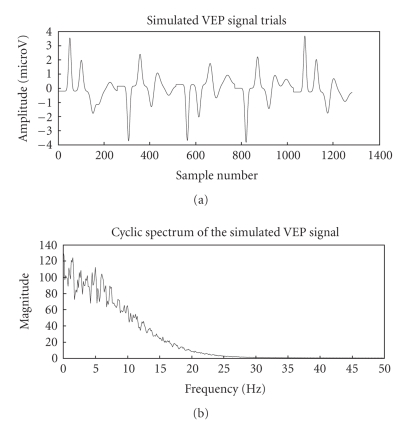
Simulated VEP signal and its cyclic spectrum.

To help study the cyclostationary property, we emulated the VEP and EEG signals that were similar to real-signal recordings. Gaussian waveforms were chosen to emulate the real-VEP-signal components as in a previous study [32] due to their suitability. The Gaussian waveform equation is given below [32]:

(9) $$G(n)=[\frac{A}{\sqrt{2\pi \sigma^{2}}}]\exp(-\frac{{\left(n-\mu \right)}^{2}}{2\sigma^{2}}),$$

where *μ* is the mean, *σ* is the standard deviation, and *A* is the amplitude of the signal. Variability between trials of the VEP signals was achieved by varying *μ*, *σ*, and *A* for the Gaussian waveforms. The simulated VEP signal and its cyclic spectrum are shown in Figure 2. The cyclic spectrum which exploits the inter trial similarities in the frequency domain depicts the cyclic VEP components at 0–10 Hz as Figure 2. In the experimental study section, similar fact is ascertained with other datasets.

**Figure 3:**
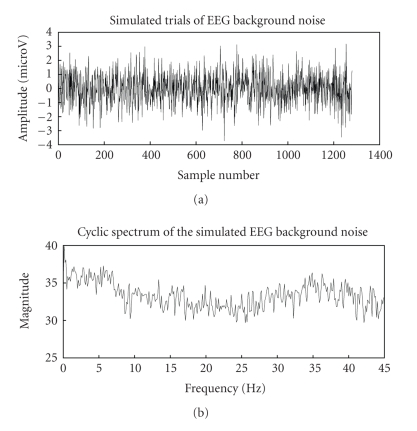
Simulated background EEG noise and its cyclic spectrum.

The stationarity of the background EEG noise has been reported in the literature [33] for periods of several hundred milliseconds. The EEG was constructed using whitening method and the AR model [34], which is as follows. Several real-EEG-signals, extracted while the subjects are at rest, were first whitened to remove correlation between their components to achieve unit variance and zero mean. Common whitening method based on the eigenvalue decomposition of the covariance matrix was used [32]. AR coefficients are then obtained from the whitened EEG signal. These AR coefficients are used for the generation of simulated background EEG noise. The simulated EEG signal and its cyclic spectrum are shown in Figure 3.

Since the background EEG noise is not cyclostationary, the cyclic spectrum is approximately flat for *f* ≠ 0 as discussed in the above cyclo model signal analysis section, which seems to justify the earlier assumed fact about the stationarity of the background EEG. Also, an important fact that the magnitude of cyclic VEP components is much more appreciable in the 0–10 Hz range than that of the background EEG is inferred from the cyclic spectrums of emulated VEP and stimulated EEG signals.

**Figure 4:**
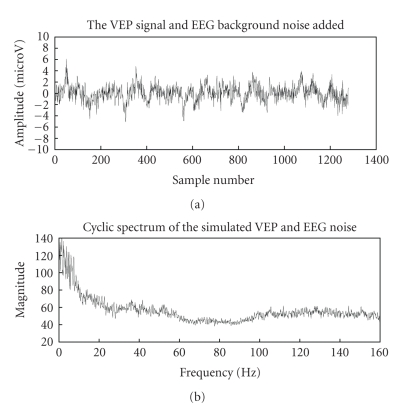
VEP with background EEG noise and its cyclic spectrum.

Additive noise assumption is usually made by all VEP estimation algorithms since there is no clear evidence in literature to suggest the nonlinear interaction of the noise and signal components. The cyclic spectrum of VEP signal with EEG noise in Figure 4 clearly highlights the cyclic VEP components. The similar magnitude spectra in the 0–10 Hz range in Figures 2 and 4 along with the discussed model seems to verify that cyclostationary model is suitable for VEP analysis.

**Figure 5:**
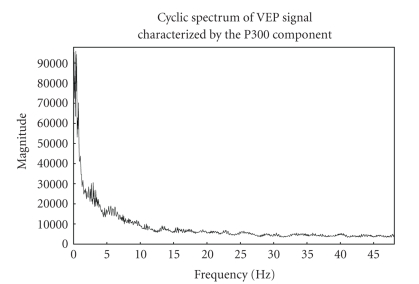
Cyclic spectrum of dataset IIb (BCI competition III).

To affirm the simulations and the discussed model, we further tested the cyclostationary algorithm with the BCI competition III (dataset IIb) provided by Wadsworth Centre, NYS Department of Health. Channel (Cz) of the training data from subject A was used to test the proposed algorithm. Figure 5 depicts the obtained cyclic spectrum of the VEP characterized by the P300 component for a character. It clearly depicts the delta (0–4 Hz) and theta (4–10 Hz) ranges as the main components of power in frequency domain for P300 waves [35, 36]. Thus, it is possible to identify the embedded endogenous components of the ERP signal with varying latency jitters in P300-based BCI systems.

A lowpass or bandpass filter can be designed based on the observed cyclic spectrum to filter the nonoverlapping EEG background noise from VEP signals for different experimental conditions and various subjects.

### 2.2. In-band denoising using genetic algorithm and mutual information

This section explores an information-theory-based approach using MI to remove the in-band EEG artifacts for VEP signal applications. It involves a variation of our previous work which makes use of G-ICA [25]. Techniques to reduce noise like adaptive filtering, ICA, and wavelets have been proposed in literature [14, 15]. ICA is a statistical method which transforms an observed multicomponent dataset into independent components that are statistically as independent as possible. For better removal of artifacts, the estimated components should be least dependent on each other. We can use measures like kurtosis, negentropy, and MI to evaluate the independence among the estimated sources [37]. In terms of robustness, cumulant-based estimators (like kurtosis) are not optimal. The main reasons are: higher-order cumulant measure the tails of the distributions, and are not influenced by structure in the middle of the distribution; the estimators of the higher order cumulants are very sensitive to outliers [37]. Their value can depend on the outliers alone. Among these various measures, MI seems to be the best choice to measure the independence of the estimated sources. MI is a measure of general dependence between two random variables [38]. Given two random variables *X* and *Y*, the mutual information *I*(*X*; *Y*) is defined as follows:

(10) I(X;Y)=H(X)+H(Y)−H(X,Y),

where *H*(⋅)denotes the entropy of random variable and measures the uncertainty associated with it. Since the EEG data is discrete we can define *H*(*X*) as follows:

(11) $$H(X)=-\sum p(X)\log_{2}p(X),$$

where *p*(*X*) represents the marginal probability distribution of the data. Mutual information has a maximum value when two time series are exactly same. The MI between random variables (here the components of the VEP signal with EEG artifacts after ICA decomposition) was estimated.

ICA seems to be the most successful of all methods to obtain independent components. Here, we present a variation from our recent work [25] using GA that minimises the MI of the extracted components to reduce the overlapping EEG noise. The mixing matrix is iteratively improved for EEG artifact separation where MI is used as the fitness function to be minimised by the GA. ICA aims at finding linear projections of the data that maximise their mutual independence [39]. It is a technique which exploits higher-order statistics and optimisation techniques for obtaining independent sources, *S* from their linear mixtures, *X*, when neither the original sources nor the actual mixing matrix *A* are known as shown below in (12) [39]. The illustration of the mathematical model is given as:

(12) $$X=AS\to \hat{S}=WX.$$

To delve deep into the method, let us consider an example. Assuming 5 trials of recordings as shown below:

(13) $$\left[\begin{array}{c} X_{1}\\ X_{2}\\ X_{3}\\ X_{4}\\ X_{5} \end{array}\right]=\left[\begin{array}{ccccc} a_{11} & a_{12} & a_{13} & a_{14} & a_{15}\\ a_{21} & a_{22} & a_{23} & a_{24} & a_{25}\\ a_{31} & a_{32} & a_{33} & a_{34} & a_{35}\\ a_{41} & a_{42} & a_{43} & a_{44} & a_{45}\\ a_{51} & a_{52} & a_{53} & a_{54} & a_{55} \end{array}\right]\left[\begin{array}{c} {\text{VEP}}_{\text{signal}}\\ {\text{EEG}}_{\text{signal}}\\ {\text{EEG}}_{\text{signal}}\\ {\text{EEG}}_{\text{signal}}\\ {\text{EEG}}_{\text{signal}} \end{array}\right].$$

It is known that in ICA methods, the task is to obtain the matrix [*W*] as in (12) to reconstruct the source matrix $\hat{S}$ as below:

(14) $$\left[\begin{array}{c} \text{VEP}+{\text{EEG}}_{1}\\ \text{VEP}+{\text{EEG}}_{2}\\ \text{VEP}+{\text{EEG}}_{3}\\ \text{VEP}+{\text{EEG}}_{4}\\ \text{VEP}+{\text{EEG}}_{5} \end{array}\right]=\left[\begin{array}{ccccc} w_{11} & w_{12} & w_{13} & w_{14} & w_{15}\\ w_{21} & w_{22} & w_{23} & w_{24} & w_{25}\\ w_{31} & w_{32} & w_{33} & w_{34} & w_{35}\\ w_{41} & w_{42} & w_{43} & w_{44} & w_{45}\\ w_{51} & w_{52} & w_{53} & w_{54} & w_{55} \end{array}\right]\left[\begin{array}{c} X_{1}\\ X_{2}\\ X_{3}\\ X_{4}\\ X_{5} \end{array}\right].$$

We then have: $\hat{S}=\text{Components of}\;(\text{VEP}+\text{EEG})$  

(15) $$\begin{array}{ll} \text{Component of}\;(\text{VEP}+{\text{EEG}}_{1})\;\text{signal} & \\ \;=W_{11}X_{1}+W_{12}X_{2}+W_{13}X_{3}+W_{14}X_{4}+W_{15}X_{5} & \\ \;\;\;\;\vdots & \\ \text{Component of}\;(\text{VEP}+{\text{EEG}}_{5})\;\text{signal} & \\ \;=W_{51}X_{1}+W_{52}X_{2}+W_{53}X_{3}+W_{54}X_{4}+W_{55}X_{5}. & \end{array}$$

G-ICA is an attractive alternative to current ICA techniques and in this proposed method, the entire matrix [*W*], will be reconstructed as GA iterates minimising the MI between the VEP signals and EEG artifacts. MI was calculated based on entropy estimates from *k* -nearest neighbour distances since they are data efficient, adaptive, and have minimal bias [40]. The mixing matrix is iteratively improved for source separation using decrements in MI which is used as the fitness function to be minimised by the GA. GA is explained using (14) and (15). GA operates on the coding of parameters rather than the parameter itself. These parameters are called chromosomes and are a string of values which represent potential solutions to the given problem. Binary chromosomes converted to realvalues represent the mixing matrix that iterates through the GA operators: selection, crossover, mutation, and inversion minimising the fitness function given by the MI between the components. Genes (bits) is used to represent each of the coefficients in [*W*] as in (15). Since 5 signals are assumedly observed as in (13) and 6bits are used for each coefficient, then each chromosome will have 150bits. A population will consist of a certain number of chromosomes; say 20, as used for this study. The gene values in the chromosomes of the initial population are randomly set for each component. These bit-valued genes are converted to realvalued in the range of [ *0,1* ]. Next, these 150 realvalued gene values are used in (15) to generate five components and then MI between the components is computed which is minimised over 100 generations to separate the in-band EEG artifacts and VEP signals.

Next selection (reproduction) is performed based on these fitness values, here, the MI between the components. During this phase of GA, chromosomes are selected from the population and recombined, producing offspring chromosomes that form the population for next generation. GA starts with an initial population and applies selection randomly from the initial population using a scheme that favours the more fit individuals (usually evaluated using the fitness function) to create the intermediate population. Good and fit chromosomes will probably be selected several times in a generation while the poor ones may not be selected at all. The common methods for performing the parent selection process are roulette wheel selection, elitist selection, and rank-based methods such as tournament selection. All three selection operators are used in this work. In tournament selection, certain numbers of chromosomes are picked randomly (in this case, 5) and the best chromosome (i.e., with the highest fitness) is stored. Since 35% of the new population will be selected using this method, this procedure is repeated to obtain 7 chromosomes, where there maybe more than one similar chromosome. Tournament selection is naturally inspired and has advantages like: absence of premature convergence and it also does not require explicit fitness function. Another 35% of the new population is selected using the roulette-wheel method. In this method, the fitness values of each chromosome are cumulatively added into a roulette wheel and when the wheel spins, there are more chances for chromosomes with higher fitness to get selected. A random number is generated to represent the wheel spin and the particular chromosome with the cumulative fitness range denoted by the number will be selected. Like in tournament selection, this is repeated 7 times to add to the existing 7 chromosomes. Rest of the population (30%) is selected using the elitist selection. In elitist selection, a number of best individuals in the population are always passed onto the next generation and this type of selection has the advantage of guaranteed convergence. Even though reproduction increases the percentage of better fitness chromosomes, the procedure is considerably sterile; it cannot create new and better chromosomes. This function is left over to crossover, mutation, and inversion operators. These operations are performed in a similar way as in our previous work [25]. Table 1 summarises the used GA parameters for this study.

## 3. EXPERIMENTAL STUDY AND RESULTS

The proposed framework to reduce background EEG noise from VEP signals was tested with BCI competition III (dataset IIb) and the P300 datasets of a subject recorded at BCI lab, University of Essex. Only a single channel (Cz) was used with 5 trials to detect the target.

### 3.1. BCI competition III (dataset IIb)

**Figure 6:**
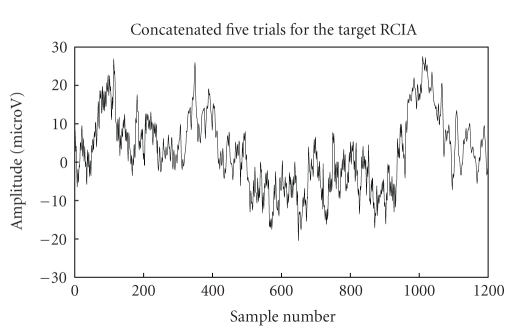
Signal trials for target RCIA from BCI competition III (dataset IIb).

**Figure 7:**
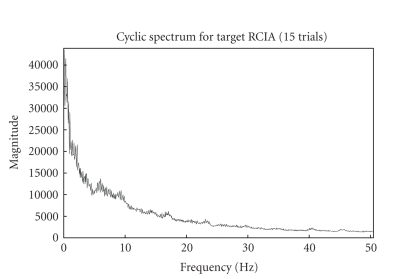
Fifteen-trials cyclic spectrum for target RCIA (just to illustrate similarity with five-trials).

**Figure 8:**
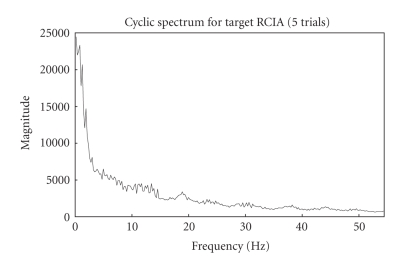
Five-trials cyclic spectrum for target RCIA.

**Figure 9:**
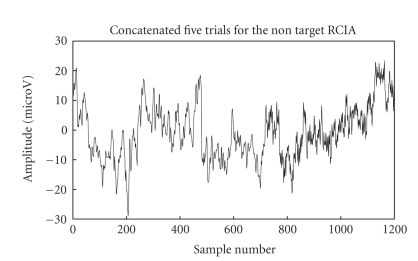
Signal trials for nontarget RCIA from BCI competition III (dataset IIb).

**Figure 10:**
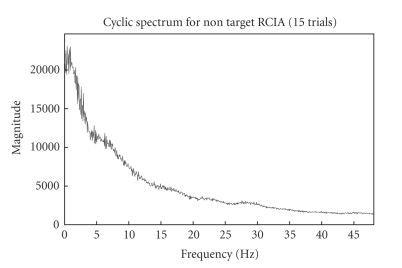
Fifteen-trial cyclic spectrum for nontarget RCIA.

**Figure 11:**
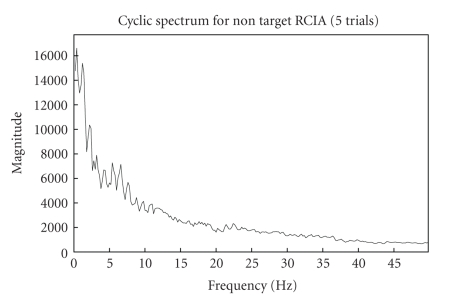
Five-trials cyclic spectrum for nontarget RCIA.

This dataset allowed a subject to communicate one of the 36 symbols presented on a 6 × 6 matrix. The dataset had specifications of 36 classes, 64 EEG channels (0.1–60 Hz), 240 Hz sampling rate, 85 training, and 100 test trials, recorded with the BCI2000 system. It followed the standard procedure developed by Farwell and Donchin for P300-based BCIs. The method assumes that the EEG epoch associated with the relevant column and the relevant row will contain a detectable P300 for a single intensification, while the other epochs will not. The data presented to our framework were obtained by averaging together each combination of row and column single-trial epochs. Thus, there were 6 rows by 6 columns = 36 row-column intersection average (RCIA). The relationship between the number of trials required and the speed of communication is direct. If detection could be achieved using just less trials, the system would allow communication at a better rate. We tested the framework using only 5 trials from channel Cz to detect “I” which is the chosen target character in the above chosen dataset. With respect to the target character “I” detection, we discuss the proposed framework's performance diagrammatically below. Figures 6 and 9 show the concatenated trials (target and nontarget RCIA) used for cyclostationary analysis while Figures 7–8 and 10–11 show their corresponding cyclic spectrums for varying number of trials.

The cyclic spectrum which exploits the inter trial similarities in the frequency domain depicts the cyclic VEP components at 0–10 Hz as in Figure 2. It can also be inferred that enhanced and better spectrum is obtained for more number of trials. The lag parameter for cyclostationary analysis was set to length of data to obtain a better spectrum. After some preliminary experimentation, five-trial cyclic spectrum was selected as optimum for analysis as it seemed to highlight the VEP signal band appreciably. A threshold for the magnitude of the cyclic spectra was used to obtain the VEP signal frequency band of (0–10 Hz) for lowpass filtering. Based on this obtained band from cyclostationary analysis, the five-trials are lowpassed-filtered using an 11th-order Chebyshev digital filter with a 3-dB cut-off frequency at 10 Hz because P300 responses are limited to this frequency range. Order 11 was used since it was sufficient to give a minimum attenuation of 60 dB in the stop band. To avoid phase distortion forward, and reverse filtering were performed since Chebyshev is a nonlinear filter. The out of band EEG artifacts is thus removed using cyclostationary analysis.

**Figure 12:**
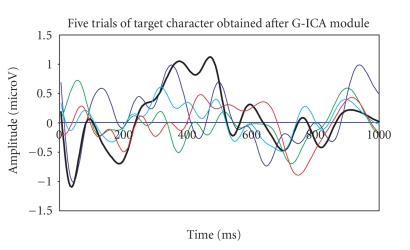
Detected P300 component for target RCIA showing higher peak amplitude.

**Figure 13:**
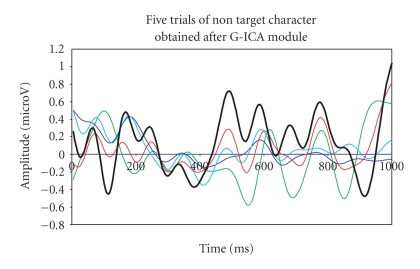
Detected P300 component for nontarget RCIA showing lower peak amplitude.

The lowpass filtered five-trials (target and nontarget RCIA) are then passed to the G-ICA fusion module to separate the in-band EEG artifacts. As discussed before, the G-ICA module works by minimising the MI of the extracted components (for 100 generations) to reduce overlapping EEG artifacts. The obtained denoised P300 response for target and nontarget cases is shown in Figures 12, 13. The P300 amplitudes for target RCIA trials were found to have a higher-peak amplitude value than that for the nontarget RCIA trials. The single trial with maximum P300 amplitude (in the range 300–600 ms) is highlighted with an increased line width in both figures.

### 3.2. BCI labs, Essex dataset

**Figure 14:**
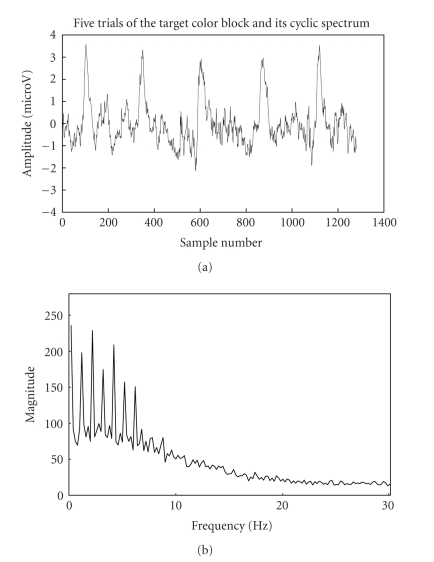
Trials for target colour block and its cyclic spectrum.

**Figure 15:**
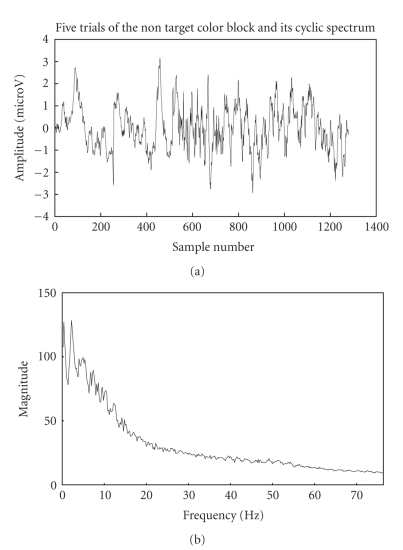
Trials for nontarget colour block and its cyclic spectrum.

**Figure 16:**
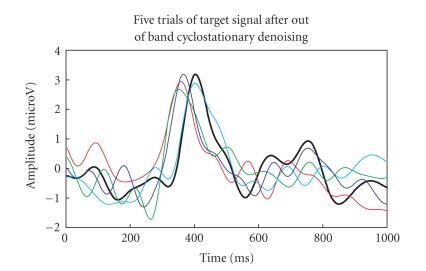
Lowpassed-filtered trials for target colour block.

**Figure 17:**
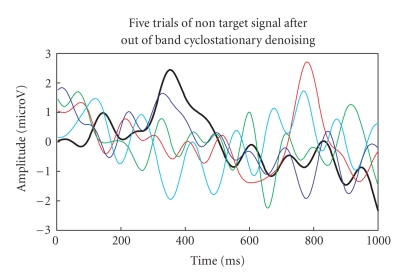
Lowpassed-filtered trials for nontarget colour block.

The presented framework was also tested offline from a dataset for a biometric application. Similar to the Donchin paradigm, the application had seven blocks of colours which were flashed to evoke P300 components. Sequences were block randomised, which means, after seven flashes each colour was flashed once, after fourteen flashes each colour was flashed twice. Forty trials were recorded (each trial had 7 flashes of the colour block). The subject was asked to focus on a single-colour block (say red) and also keep a count of the number of times it flashed, which enabled monitoring the performance of the subject. The colour blocks were flashed for 100 millisecond with an interstimulus interval of 300 millisecond. EEG recordings were carried out on a Biosemi Active Two system using 34 channels (32 on a scalp and 2 on either mastoids); however, only channel Cz was used. Data was sampled at 256 Hz with no filtering. The subject was a male aged 27 who had experience of using the BCIs before, with no known neurological disorders. The performance of the framework for target and nontarget color blocks is discussed below diagrammatically. It can be seen from Figure 14 that the target-trial data is cyclic in time domain and also that the magnitude of the cyclic spectrum is much higher than that of the nontarget data as in Figure 15. The lag parameter for cyclostationary analysis was set to length of data. After some preliminary experimentation, five-trial cyclic spectrums were again found to be optimum for analysis as it seemed to highlight the VEP signal band appreciably. A threshold for the magnitude of the cyclic spectra was used to obtain the VEP signal frequency band of (0–10Hz) for lowpass filtering. Based on this obtained band from cyclostationary analysis, a lowpass filter for (target colour block and nontarget colour block) was used as in Section 3.1 to remove nonoverlapping EEG artifacts and the output is shown in Figures 16-17.

**Figure 18:**
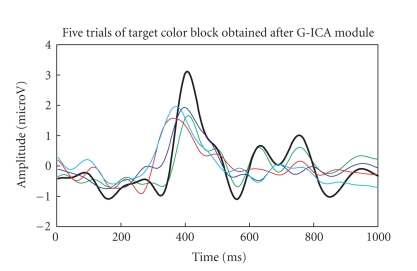
P300 components of the five-trials for target colour block using G-ICA.

**Figure 19:**
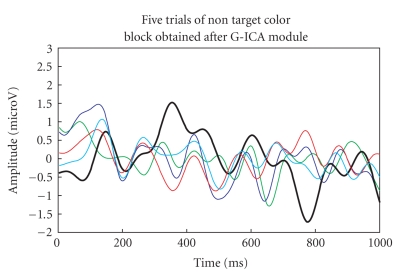
P300 components of the five-trials for nontarget colour block using G-ICA.

The five-trials (target colour block and nontarget colour block) are then passed to G-ICA fusion module. Investigating Figures 18, 19 clearly shows that the P300 component amplitude in the 300–600 millisecond range is higher for target-colour block than the nontarget colour block. The single trial with maximum P300 amplitude is highlighted with an increased line width in both the figures. It was also observed that the frequency band (CS analysis) and the unmixing matrix (G-ICA) do not change over trials.

**Figure 20:**
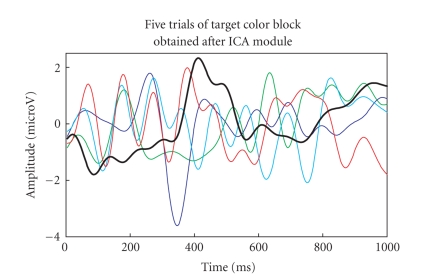
P300 components of the five-trials for target colour block using ICA.

**Figure 21:**
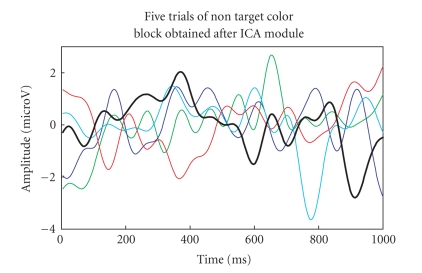
P300 components of the five-trials for nontarget colour block using ICA.

We also compared the performance of G-ICA with ICA (fixed point-ICA). The five-trials (target colour block and nontarget colour block) after lowpass filtering, when passed through ICA module gave the outputs as depicted in Figures 20-21. Again, the single trial with maximum P300 amplitude (300–600 ms) is highlighted with an increased line width in both the figures. It can be observed from Figures 18–21 that the threshold of difference between target and nontarget for G-ICA is higher than that obtained using ICA. Comparison in terms of runtime in seconds is indicated in Table 2 and it was found to be comparable.

## 4. DISCUSSION AND CONCLUSION

A new framework for enhanced VEP signal detection is presented. The two-stage framework makes use of cyclostationary and G-ICA techniques to separate VEP signals from EEG artifacts. Brain signals were emulated using VEP contaminated with EEG in the simulation study to analyse the cyclo model for brain signal analysis. Studies from this work seem to suggest that cyclostationary model might be suitable for VEP signal analysis. To validate the method, further the algorithms were tested to identify an arbitrarily chosen character “I” from the BCI competition III challenge (dataset IIb) and also with datasets recorded at BCI lab, University of Essex which gave satisfactory results with very few trials (5 trials). The G-ICA fusion module does not assume any property of noise hence it can be used to separate any type of linear additive noise. The runtime performance of G-ICA and ICA was similar and comparable. It was also observed that the frequency bands and unmixing matrix do not change over trials for a given subject; hence the CS and G-ICA methods need to be applied only to training data. It is known that in a P300-based BCI system the communication speed of characters is dependent on the number of trials. Hence, this proposed signal preprocessing framework may be used to reduce the number of trials and thereby increase the rate of communication.

## Figures and Tables

**Table 1:** Parameters for genetic algorithm.

Coding of genes	Binary coding converted to real value [*1,0*] for fitness computation
Fitness function	Mutual information (MI)
Population size	20
no of genes	6 bits for each gene
Reproduction	Elitist selection (30% of population), tournament selection
	(35% of population), and roulette selection (35% of population)
Crossover type and rate	Uniform crossover, 0.5
Mutation type and rate	Randomly mutate selected bits, 0.01
Inversion type and rate	Inversion between 2 randomly selected points, 0.01
Convergence	100
Repetition	3

**Table 2:** Runtime comparison between G-ICA and ICA.

	G-ICA	ICA
Target color block	97.23 s	96.16 s
Nontarget color block	98.03 s	97.05 s

## References

[1] Misulis KE (1994). *Sphelmann's Evoked Potential Primer: Visual, Auditory, and Somatosensory Evoked Potentials in Clinical Diagnosis*.

[2] Aunon JI, McGillem CD, Childers DG (1981). Signal processing in event potential research: averaging and modelling. *CRC Critical Reviews in Bioengineering*.

[3] Lange DH, Siegelmann HT, Pratt H, Inbar GF (2000). Overcoming selective ensemble averaging: unsupervised identification of event-related brain potentials. *IEEE Transactions on Biomedical Engineering*.

[4] Handy CT (2005). *Event-Related Potentials: A Methods Handbook*.

[5] Kutas M, McCarthy G, Donchin E (1977). Augmenting mental chronometry: the P300 as a measure of stimulus evaluation time. *Science*.

[6] Donchin E, Ritter W, McCallum C, Callaway E, Tueting P, Koslow SH (1978). Cognitive psychophysiology: the endogenous components of the ERP. *Brain Event-Related Potentials in Man*.

[7] Coles MGH, Rugg MD, Rugg MD, Coles MGH (1995). Event-related brain potentials: an introduction. *Electrophysiology of Mind: Event-Related Brain Potentials and Cognition*.

[8] Donchin E, Spencer KM, Wijesinghe R (2000). The mental prosthesis: assessing the speed of a P300-based brain-computer interface. *IEEE Transactions on Rehabilitation Engineering*.

[9] Serby H, Yom-Tov E, Inbar GF (2005). An improved P300-based brain-computer interface. *IEEE Transactions on Neural Systems and Rehabilitation Engineering*.

[10] Farwell LA, Donchin E (1988). Talking off the top of your head: toward a mental prosthesis utilizing event-related brain potentials. *Electroencephalography and Clinical Neurophysiology*.

[11] Woody CD (1967). Characterization of an adaptive filter for the characterization of variable latency neuroelectric signals. *Medical and Biological Engineering and Computing*.

[12] Wastell DG (1977). Statistical detection of individual evoked responses: an evaluation of Woody's adaptive filter. *Electroencephalography and Clinical Neurophysiology*.

[13] Jung T-P, Makeig S, Westerfield M, Townsend J, Courchesne E, Sejnowski TJ (2001). Analysis and visualization of single-trial event-related potentials. *Human Brain Mapping*.

[14] Makeig S, Westerfield M, Jung T-P (1999). Functionally independent components of the late positive event-related potential during visual spatial attention. *Journal of Neuroscience*.

[15] Drozd M, Husar P, Nowakowski A, Henning G (2005). Detecting evoked potentials with SVD- and ICA-based statistical models. *IEEE Engineering in Medicine and Biology Magazine*.

[16] Moser JM, Aunon JI (1986). Classification and detection of single evoked brain potentials using time-frequency amplitude features. *IEEE Transactions on Biomedical Engineering*.

[17] Gevins AS, Morgan NH, Bressler SL, Doyle JC, Cutillo BA (1986). Improved event-related potentials estimation using statistical pattern classification. *Electroencephalography and Clinical Neurophysiology*.

[18] Zouridakis G, Jansen BH, Boutros NN (1997). A fuzzy clustering approach to EP estimation. *IEEE Transactions on Biomedical Engineering*.

[19] Tong L, Xu G, Kailath T (1994). Blind identification and equalization based on second-order statistics: a time domain approach. *IEEE Transactions on Information Theory*.

[20] Koenig D, Boehme J Application of cyclostationary and time-frequency signal analysisto car engine diagnosis.

[21] Ohno S, Sakai H (1996). Optimization of filter banks using cyclostationary spectral analysis. *IEEE Transactions on Signal Processing*.

[22] Gardner WA (1990). Identification of systems with cyclostationary input and correlated input/output measurement noise. *IEEE Transactions on Automatic Control*.

[23] Gardner WA (1991). Exploitation of spectral redundancy in cyclostationary signals. *IEEE Signal Processing Magazine*.

[24] Gardner WA (1988). Signal interception: a unifying theoretical framework for feature detection. *IEEE Transactions on Communications*.

[25] Palaniappan R, Gupta CN Genetic algorithm based independent component analysis to separate noise from Electrocardiogram signals.

[26] Zeng X-Y, Chen Y-W, Nakao Z, Yamashita K (2000). Signal separation by independent component analysis based on agenetic algorithm.

[27] Goldberg DE (1989). *Genetic Algorithms in Search, Optimization and Machine Learning*.

[28] Rojas F, Puntonet CG, Rodríguez-Álvarez M, Rojas I, Martín-Clemente R (2004). Blind source separation in post-nonlinear mixtures using competitive learning, simulated annealing, and a genetic algorithm. *IEEE Transactions on Systems, Man and Cybernetics*.

[29] Wang J, Chen T, Huang B (2006). Cyclo-period estimation for discrete-time cyclo-stationary signals. *IEEE Transactions on Signal Procesing*.

[30] Giannakis GB (1999). Cyclo-stationary signal analysis. *Digital Signal Processing Handbook*.

[31] Paliwal KK, Sagisaka Y Cyclic autocorrelation-based linear prediction analysis of speech.

[32] Sharmilakanna P, Palaniappan R EEG artifact reduction in VEP using 2-stage PCA and N4 analysis of alcoholics.

[33] McEwen JA, Anderson GB (1975). Modeling the stationarity and gaussianity of spontaneous electroencephalographic activity. *IEEE Transactions on Biomedical Engineering*.

[34] Karjalainen PA, Kaipio JP, Koistinen AS, Vauhkonen M (1999). Subspace regularization method for the single-trial estimation of evoked potentials. *IEEE Transactions on Biomedical Engineering*.

[35] Klimesch W (1995). The P300 wave and band power in the alpha and theta range. *Psycoloquy*.

[36] Verleger R (1995). Memory-related EEG potentials: slow negativities, priming positivity, recognition positivity, and Dm. *Psycoloquy*.

[37] Hyvarinen A, Oja E (1999). A survey on independent component analysis. *Neural Computing Surveys*.

[38] Scott DW (1992). *Multivariate Density Estimation: Theory, Practice, and Visualization*.

[39] Comon P (1994). Independent component analysis, a new concept?. *Signal Processing*.

[40] Kraskov A, Stögbauer H, Grassberger P (2004). Estimating mutual information. *Physical Review E*.

